# Functional polymorphisms in pigmentation-related genes *MC1R* and *DCT* display population-specific association with wet age-related macular degeneration

**DOI:** 10.1016/j.bbrep.2026.102477

**Published:** 2026-02-06

**Authors:** Mika Reinisalo, Seppo Helisalmi, Ali Koskela, Jenni Küblbeck, Mikko Liukkonen, Maija Mutikainen, Angela J. Cree, Helen Griffiths, Andras Papp, Sanna Seitsonen, Ilkka Immonen, Hilkka Soininen, Arto Urtti, Mikko Hiltunen, Mateusz Winiarczyk, Miklos Resch, J. Arjuna Ratnayaka, Andrew J. Lotery, Kai Kaarniranta, Paavo Honkakoski

**Affiliations:** a School of Pharmacy, Faculty of Health Sciences, University of Eastern Finland, P.O.Box 1627, Kuopio, FI-70211, Finland; b Institute of Clinical Medicine, Internal Medicine, University of Eastern Finland, Kuopio, Finland; c Department of Neurology, School of Medicine, Faculty of Health Sciences, University of Eastern Finland, P.O.Box 1627, Kuopio, FI-70211, Finland; d Department of Ophthalmology, Helsinki University Hospital, HUS, P.O.Box 220, Helsinki, 00029, Finland; e Institute of Biomedicine, School of Medicine, Faculty of Health Sciences, University of Eastern Finland, P.O.Box 1627, Kuopio, FI-70211, Finland; f Department of Vitreoretinal Surgery, Medical University of Lublin, Lublin, 20-079, Poland; g Department of Ophthalmology, Semmelweis University, Budapest, 1085, Hungary; h Clinical and Experimental Sciences, Faculty of Medicine, University of Southampton, Southampton, SO17 1 B, UKJ, UK; i Department of Ophthalmology, School of Medicine, Faculty of Health Sciences, University of Eastern Finland, P.O.Box 1627, Kuopio, FI-70211, Finland; j Department of Ophthalmology, Kuopio University Hospital, KYS, Kuopio, 00029, Finland; k Division of Pharmacotherapy and Experimental Therapeutics, Eshelman School of Pharmacy, University of North Carolina at Chapel Hill, NC, 27599, USA

**Keywords:** Wet age-related macular degeneration, Pigmentation, Dopachrome tautomerase, Melanocortin-1 receptor, Polymorphism

## Abstract

Age-related macular degeneration (AMD) is among the leading causes of vision loss. Factors increasing the risk of AMD include aging, smoking, cardiovascular diseases and heritability. Although melanin pigment is known to protect retinal homeostasis, the link between pigmentation-related genes and AMD is unclear. We investigated associations between 26 variations in six pigmentation-related genes and wet AMD risk in a Finnish population, followed by replication in the United Kingdom (UK), Hungarian and Polish cohorts, totaling 775 patients and 959 controls. Associations of genetic components with smoking and body mass index (BMI) were tested in the Finnish and UK cohorts. The functionality of candidate variants in human retinal pigment epithelial (RPE) cells was evaluated using gene promoter analysis and gene silencing.

Non-coding variants, rs1407995 in the dopachrome tautomerase (*DCT*) intron and rs3212351 in the melanocortin-1 receptor (*MC1R*) promoter, were associated with wet AMD in the Finnish cohort. The variant rs3212351 disrupts a binding site for transcription factor MITF and reduces MC1R expression in RPE cells. Unlike in the Finnish cohort, the data regarding the MC1R variant suggested a protective association in the Polish cohort. The incidence of AMD increased with age in all cohorts. Smoking increased AMD risk in the cohorts studied. Sex and BMI showed no associations.

These findings suggest that variations in *DCT* and *MC1R* genes known to affect skin and eye pigmentation may also play a role in development of wet AMD. The observed population differences may be related to variable pigmentation traits.

## Highlights

• Variations in pigmentation genes Dopachrome Tautomerase (DCT) and Melanocortin receptor 1 (MC1R) are associated with wet form of age-related macular degeneration (wet AMD).
• Studied non-coding variants rs1407995 in the DCT intron and rs3212351 in the MC1R promoter are functional.
• The observed population differences may associate with variable pigmentation traits.

## Graphical abstract

**Figure:**
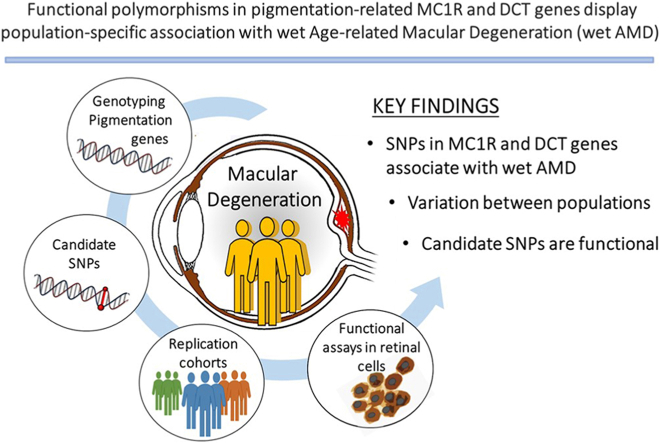

## 1 Background

Age-related macular degeneration (AMD) is the most frequent cause of blindness in developed societies [1]. Choroidal neovascularization with subretinal bleeding is a clinical hallmark for wet AMD, whereas progressive retinal atrophy with drusen accumulation is typical for dry AMD. Chronic oxidative stress, impaired proteostasis and inflammation play a prominent role in AMD pathogenesis. Dysfunction of the retinal pigment epithelium (RPE), which supports the overlying photoreceptors, as well as changes to associated tissues such as the Bruch's membrane and choroid, results in the loss of central vision [2,3]. The most significant risk factors for AMD include age, heritability, smoking and cardiovascular diseases [1]. Epidemiological studies suggest that changes in retinal pigmentation and lighter eye and hair color may be associated with different forms of AMD [1,4]. Experiments indicate that low eumelanin pigment levels are associated with retinal degeneration in mice [5] and an inability to alleviate oxidative stress in RPE cells [6]. In addition, melanin and its precursor L-3,4-dihydroxyphenylalanine (l-DOPA) are shown to be important for retinal development and vision in murine models [7,8]. Administration of l-DOPA to patients can significantly delay the development of wet AMD [9].

The eye is composed of several heavily pigmented tissues, such as the iris and choroid, which form the uveal layer, as well as the RPE layer beneath the photoreceptors. Like all pigmented cells, RPE cells synthesize and deposit melanin in intracellular melanosomes, which protect retinal cells from UV irradiation [10]. Melanin also protects against oxidative stress due to its capacity to scavenge free radicals and sequester free radical-forming metal ions [11]. The molecular mechanisms of melanogenesis have primarily been studied in skin melanoma cells. The principal regulator is the melanocortin-1 receptor (MC1R), a G protein-coupled receptor activated by neuropeptides α-melanocyte stimulating hormone (αMSH) and adrenocorticotrophin [12]. Activated MC1R modulates intracellular cAMP levels, thereby enhancing expression of the microphthalmia-associated transcription factor (MITF) [13] that is essential for RPE development. In turn, MITF increases transcription of several genes involved in melanin synthesis such as tyrosinase (TYR*)*, tyrosinase-related protein 1 (TYRP1) and dopachrome tautomerase (DCT) [14]. Specifically, in the RPE, orthodenticle homeobox-2 (OTX2) transcription factor synergizes with MITF in activating genes of the *TYR* family [15].

Although biological studies support defective pigmentation as a critical factor for retinal disorders, the link between genetic variation within the pigmentation-related genes and AMD is unclear. Large-scale genome-wide association studies (GWAS) on advanced AMD have mainly identified candidate genes involved in complement activation, lipid transport and extracellular matrix formation [16]. In contrast, polymorphisms in *TYR*, *TYRP1*, *DCT* and *MC1R* genes are strongly associated with natural phenotypic variations in skin pigmentation [17]. For example, MC1R variants that reduced synthesis of black/brown melanin result in the pheomelanin phenotype such as red/blond hair, fair skin, freckles. These individuals are also more sensitive to sun, which is likely due to poor protection by pheomelanin against oxidative damage and light exposure [18].

Evidence that genetic variations affecting skin color in different populations would also influence the levels of ocular pigmentation or RPE melanin synthesis is limited. However, recently GWAS by Gorman et al. [19] bring forth the association of different pigmentation traits and pigmentation genes (HERC2/OCA2, TYR and TRPM1) with increased AMD risk. They showed that association of pigmentation traits with AMD was more prominent in the population of European ancestry. This is in line with epidemiological studies showing that the prevalence of AMD is lower in the black population of African origin than in Caucasians [20], suggesting an association with ocular melanin levels. In addition, Weiter and colleagues showed racial differences of melanin levels in the posterior eye segment as well as decrease of RPE and choroidal melanin content with age [21]. Importantly, expression quantitative trait loci (eQTL) mapping studies have shown associations between RPE-linked diseases and functional variants in pigmentation genes such as the recently identified functional rs4547091 in the human *TYR* gene regulatory region [15,22]. This variant was connected with albinism where melanin pigment is lacking [23]. Individuals with oculocutaneous albinism often harbor loss-of-function variants in melanogenic enzymes and present with retinal malformations and reduced visual acuity [24,25]. In addition, mutations in MITF and OTX2 regulator proteins are involved in abnormalities in RPE pigmentation, severe ocular malformations and retinal degeneration [5,26].

Because of population-specific differences in AMD variants [27,28], we set out to compare the association of selected pigmentation gene variants in Finnish wet AMD patients vs. age-matched control subjects. We show here that variants in the *DCT* gene intron (rs1407995) and *MC1R* gene promoter (rs3212351) are associated with wet AMD in this cohort. The DCT and MC1R candidate variants were replicated in cohorts from the United Kingdom (UK), Hungary and Poland. Interestingly, our replication studies suggested protective association for the MC1R variant in the Polish cohort, whereas no associations were detected in the other cohorts. Moreover, functional promoter analysis indicated that rs3212351 and rs3212360 variants in the *MC1R* regulatory region disrupt binding sites for the transcription factors MITF and specificity protein-1 (Sp1), respectively, altering the MC1R activity in human RPE cells. Moreover, the essential role of MITF and Sp1 factors was demonstrated by silencing their expression, which significantly decreased the mRNA expression of the MC1R gene in RPE cells.

## 2 Methods

### 2.1 The Finnish study cohort

Finnish wet AMD patients (Supplemental Table S1) were diagnosed by fundus photographs, optical coherent tomography (OCT) and fluorescein angiography [29]. The control subjects had no signs of AMD in fundus photographs and were free from diabetes mellitus. This study followed the Declaration of Helsinki, and the use of Finnish study cohort was also approved by the Ethics Committee of the Kuopio University Hospital (approval number 42/2014).

### 2.2 Replication cohorts

Prevalence of selected SNPs was replicated in wet AMD cohorts from Poland, Hungary and the UK (Table S1). For the Polish cohort, the Bioethical Committee of Medical University of Lublin approved the study (declaration number KE-0254/238/2015). In Hungary, the present study was approved by the Hungarian Medical Research Council Committee of Science and Research Ethics (Approval No. 15028-2/2017/EKU). In the UK, consent was obtained in accordance with the Declaration of Helsinki and was approved by South West Hampshire Local Research Ethics Committee (374/02/t and 150/03/t). Informed consent was obtained from all subjects in these cohorts, and all methods were carried out in accordance with the relevant guidelines and regulations of local research ethics committees. Altogether, a total of 775 wet AMD patients and 959 control subjects were examined. The principles used for selection of study subjects in the Finnish cohort were also applied to the replication cohorts. In addition to age and gender information from all cohorts, the lifestyle and metabolic parameters such as smoking status and body mass index (BMI) were available from the Finnish and UK cohorts and taken into account. Smoking status was grouped to non-smokers (have never smoked), occasional/ex-smokers and regular smokers (long and continuing smoking history), whereas measured BMIs were determined as continuous variable.

#### 2.2.1 Genotyping

The selected genes are either directly or indirectly involved in melanin synthesis in the RPE and other pigmented tissues. We chose 26 single nucleotide polymorphisms (SNPs) within *TYR*, *TYRP1*, *DCT*, *MC1R*, *MITF* and *OTX2* genes for characterization in the Finnish cohort (Table S2). Variants were mainly located in non-coding regions of genes such as promoters, intronic and 3′-untranslated regions. The haplotype structure of genes was characterized using the Haploview program to cover all studied regions and restrict the number of variants for genotyping [30]. DNA was extracted from peripheral blood [29]. Variants were genotyped using the Sequenom iPlex platform (Sequenom, Hamburg, Germany) at UEF. Samples with an average call rate of ≥90 % were included. All variants for the association analysis conformed to the Hardy-Weinberg equilibrium.

Replication cohorts from Poland, Hungary and the UK were investigated with respect to variants rs1407995 and rs3212351. Genotyping of Polish and Hungarian cohorts was performed using TaqMan SNP Genotyping Assay ID: C_7593137_20 for variant rs1407995 and Custom Genotyping Assay for rs3212351, both provided by Thermo Fisher Scientific (Foster City, CA, USA). DNA extraction and genotyping of the UK cohort was carried out at LGC sequencing service (LGC Service Lab UK).

#### 2.2.2 Cell culture

Retinal pigment epithelial (RPE) cell lines ARPE-19 [31], D407 [32] and human primary RPE (hRPE) cells (HRPEpiC #6540; Sciencell, Carlsbad, CA) were maintained as previously described [33,34]. Cells were cultured at +37 °C and 5 % CO_2_.

#### 2.2.3 MC1R plasmid construction and site-directed mutagenesis

Human *MC1R* promoter fragment (1.7 kb; −1398 to +346) was cleaved from the pKM2L-phMC1R plasmid (RIKEN, RDB5515) by *Xho*I and *EcoR*I digestion and inserted into *Xho*I/*EcoR*I-digested pGL3-Basic (MCS+) luciferase reporter vector (Promega, Madison, WI). The resulting construct was designed as hMC1R-1398 (wildtype) (Fig. 2). The desired mutations were introduced into the *hMC1R* promoter by using the QuickChange II XL Site-Directed Mutagenesis Kit (Stratagene, La Jolla, CA) according to the manufacturer's instructions with mutagenic primers (Table S3). Mutated constructs with SNPs rs3212351 at the MITF and rs3212360 at the Sp1 binding sites were designated as hMC1R–1398SNP/M2 and hMC1R–1398SNP/S, respectively (Fig. 2). All constructs were verified with dideoxy sequencing.

#### 2.2.4 Transient transfection of promoter constructs

Functionality of the promoter constructs was assessed by polyethyleneimine 25 (PEI25) -mediated reverse transfection, followed by luciferase reporter assays [15]. ARPE-19 (1.2 × 10^5^ cells per well) and D407 (0.6 × 10^5^ cells per well) cells were cultured overnight on 48-well plates in culture medium containing 10 % FBS. DNA/PEI25 complexes [0.6 μg of *hMC1R* promoter constructs or promoterless pGL3-basic (Promega) and 0.3 μg of pCMVβ control plasmid (Clontech, Palo Alto, CA)] were delivered into the cells in serum-free medium and incubated for 5 h. After transfection, an equal volume of fresh culture medium containing 10 % FBS was added and the cells were incubated for further 48 h. The cells were lysed in 70 μl of lysis buffer and the luciferase and β-galactosidase activities assayed from 20 μl of lysate with Victor 1320 multilabel plate reader (PerkinElmer, Turku, Finland) as previously described [15]. The transfections were performed as three independent experiments, each done with three replicate wells per promoter construct.

#### 2.2.5 Transfection of small interfering RNA (siRNA)

The binding sites for Sp1 and MITF transcription factors are close to each other within the *MC1R* promoter and potentially affected by SNPs rs3212351 and rs3212360. Their roles as regulators of *MC1R* expression were studied by transfecting siRNAs specific for *Sp1* (Ambion s13319; ThermoFisher, Austin, TX) and *MITF-A* isoform (Ambion s8790) into hRPE cells at final concentration of 25 nM as described earlier [15] with the exception that siRNA transfection was performed 48 h after plating the cells on a 48-well plate (1.0 × 10^5^ cells per well). Silencer Select Negative Control #1 siRNA was used as a control.

### 2.3 RNA isolation and quantitative reverse transcription PCR

RNA isolation, DNase treatment and cDNA synthesis were carried out 48 h after siRNA transfection by using the TaqMan Gene Expression Cells-to-Ct Kit (Ambion). Primers are listed in Table S3. Quantitative PCR was performed as described earlier [13] with an ABI Prism 7500 instrument (Applied Biosystems). Threshold cycle (C_t_) values for endogenous *MC1R*, *MITF* and *Sp1* mRNAs were normalized relative to quantitated RNA levels in cDNA synthesis as described earlier [15].

### 2.4 Statistical methods

SPSS version 21.0.0.0 (Chicago, IL, USA) was used for the statistical analysis of genotyping data. For meta-analysis and forest plots, meta 5.2–0, metafor 3.4–0 and dmetar 0.0.9000 packages were used [[35], [36], [37]]. Genetic associations with wet AMD were examined using a binary logistic regression analysis which was also adjusted for age, gender, smoking status and BMI. Associations between wet AMD and these variables were also evaluated using the Pearson chi-square test for the categorical and Mann-Whitney *U* test for the continuous variables. Student's *t*-test with Bonferroni correction was used for pairwise statistical analysis of functional data. The results at *p* < 0.05 (∗) were considered statistically significant.

## 3 Results

### 3.1 The gene variants DCT rs1407995 and MC1R rs3212351 are associated with risk of wet AMD in the Finnish cohort

Out of 26 SNPs investigated in the Finnish cohort, two variants, *rs*1407995 and rs3212351 in *DCT* and *MC1R* genes, respectively, showed association with an increased risk of wet AMD when compared to controls. Association of the *DCT* variant *rs*1407995 with wet AMD was seen under a crude [OR = 3.250; 95 % CI (1.036–10.195), *p* = 4.3 × 10^−2^] as well as in age- and sex -adjusted [OR = 4.514; 95 % CI (1.300–15.669), *p* = 1.8 × 10^−2^] recessive genetic models (Table S4). The variant rs3212351 showed significant association under a crude recessive genetic model [OR = 1.714; 95 % CI (1.004–2.924), *p* = 4.8 × 10^−2^]. No other significant SNPs within the studied genes were found. Bonferroni-corrected *p*-values after multiple comparisons for 26 SNPs were naturally not significant. Data under additive and dominant genetic models are shown in the supplements (Table S5).

### 3.2 Replication studies on variants DCT rs1407995 and MC1R rs3212351

**Fig. 1:**
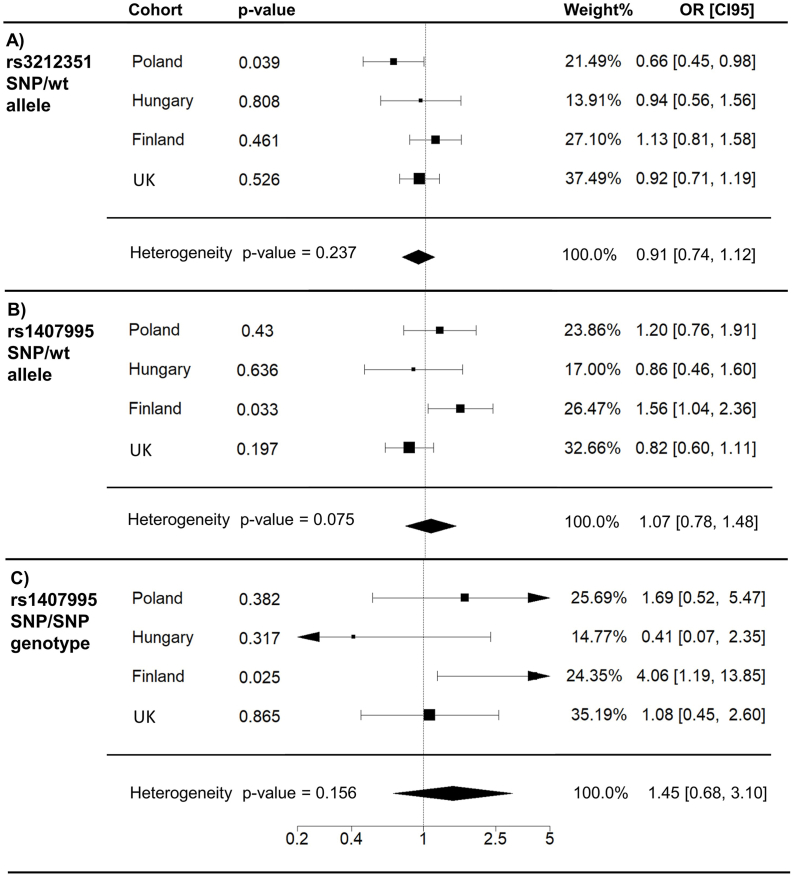
Forest plots representing allele (panel A and B) and genotype (panel C) association of DCT rs1407995 and MC1R rs3212351 variants to wet AMD in the Polish, Hungarian, Finnish and the UK cohorts. Age and sex adjusted odds ratios (OR) with 95 % confidence intervals (CI95), related *p*-values, and weight (%) of each cohort in the study are shown. Polymorphism specific alleles are shown in Table S2.

Associations of rs1407995 and rs3212351 variants that were deemed causative in the Finnish cohort were replicated in the Polish, Hungarian and UK case-control cohorts. Interestingly and in contrast to the Finnish cohort, the rs3212351 allele data proposed protective association with a decreased risk for wet AMD in the Polish cohort [Fig. 1A; OR = 0.67; 95 % CI (0.46–0.98), *p* = 3.7 × 10^−2^]. This association was also noted clearly when adjusted for age and sex [OR = 0.66; 95 % CI (0.45–0.98), *p* = 3.9 × 10^−2^], whereas in other populations the role of this variant was insignificant. In the Finnish cohort, the allele rs1407995 [Fig. 1B; OR = 1.56; 95 % CI (1.04–2.36), *p* = 3.3 × 10^−2^] and its homozygous genotype [Fig. 1C; OR = 4.06; 95 % CI (1.19–13.85), *p* = 2.5 × 10^−2^] displayed a significant increased risk for wet AMD when adjusted for age and gender. However, no association to wet AMD was observed in other cohorts. Forest plots of all cohorts displaying alternative genotypes are shown in the Supplementary Figs. S1 and S2.

**Fig. 2:**
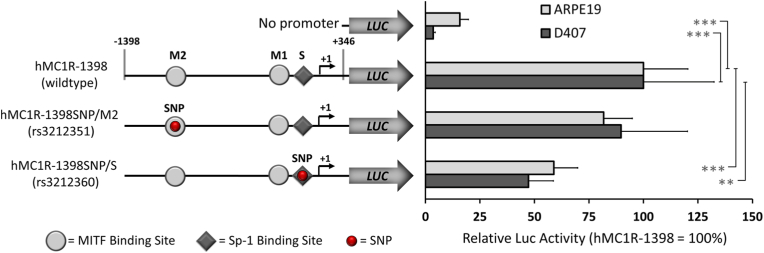
Polymorphisms decrease human MC1R promoter activity. Constructs with the ancestral (hMC1R-1398) or mutated MC1R promoters (hMC1R–1398SNP/M2 for rs3212351; hMC1R–1398SNP/S for rs3212360) or the promoterless pGL3-Basic luciferase reporter were transfected into ARPE-19 and D407 RPE cells. Ancestral transcription factor sites include two MITF sites **M1** at −116/-111 (CATGTG) and **M2** at −1236/-1231 (inverted, CACATG) and the Sp1 site (**S**) at −109/-104 (CCGCCC) indicated with symbols. Variants rs3212351 (2-bp deletion at −1233/-1232) and rs3212360 (−105C > T) are shown by circles. Normalized luciferase activities are shown relative to the ancestral MC1R promoter (=100 %). The data is presented as mean ± SD from three independent transfections, each performed in triplicate. One-tailed *t*-test with Bonferroni correction; ∗*p* < 0.05, ∗∗*p* < 0.01 and ∗∗∗*p* < 0.001.

### 3.3 Age and smoking are associated with risk of wet AMD

In all cohorts studied, aging clearly increased the incidence of wet AMD whereas the role of sex was not significant (Table S1). Information regarding smoking status and BMI was available from the Finnish and UK cohorts only. In both cohorts, the incidence of wet AMD was significantly increased due to smoking (Finland, *p* = 2.5 × 10^−2^; UK, *p* < 1.0 × 10^−3^) whereas BMI did not increase the incidence of wet AMD. However, a significant association between BMI and wet AMD together with age and smoking was observed in the Finnish cohort [OR = 1.068; 95 % CI (1.004–1.137), *p* = 3.7 × 10^−2^] (Table S8).

Furthermore, we also evaluated the associations between the risk of wet AMD, the candidate SNPs and each variable (smoking, BMI, age and gender) in Finnish and UK cohorts (Supplemental Table S6–S9). In the Finnish cohort (Table S6), the genotyping data showed associations between the polymorphism *rs*1407995, wet AMD and variables: age, gender, smoking as well as BMI [OR = 1.685; 95 % CI (0.999–2.843), *p* = 5.0 × 10^−2^] with increased incidence linked with age and smoking: OR = 1.753; 95 % CI (1.055–2.915), *p* = 3.0 × 10^−2^].

### 3.4 The human proximal MC1R promoter variants are functional

The AMD-associated polymorphism rs3212351 (AT > del) with a 2-bp deletion disrupts a potential consensus binding sequence (CACATG) at the **M2** binding element for MITF in the *MC1R* promoter (Fig. 2). Another SNP rs3212360 (−105C > T) nearby disrupts the consensus binding sequence (CCGCCC) for the transcription factor Sp1 (binding element **S**), which is very close to the known MITF binding element **M1** (CATGTG). Both binding elements **S** and **M1** are essential for *MC1R* promoter activity in melanoma cells [13,38]. To study functionality of these variants in RPE cells, SNPs were introduced into the *MC1R* promoter by site-directed mutagenesis and their effects tested by a luciferase reporter gene assay in ARPE-19 and D407 human RPE cell-lines.

In ARPE-19 and D407 cells, the 2-bp deletion in rs3212351 decreased the reporter activity slightly to 82 % and 90 % respectively, compared to the wildtype promoter (Fig. 2). When rs3212360 with the T allele was present, the activity of *MC1R* promoter was significantly reduced to 59 % and 47 % of the wildtype promoter in ARPE-19 and D407 cell lines, respectively.

### 3.5 Knockdown of endogenous MITF-A and Sp1 decreased MC1R mRNA expression in human primary RPE cells

**Fig. 3:**
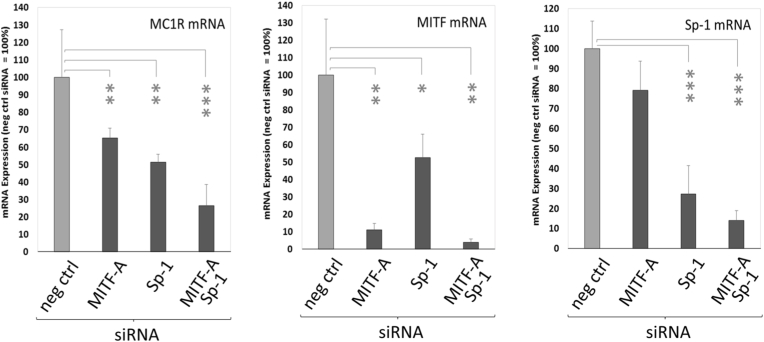
Silencing of endogenous MITF-A and Sp1 decreases MC1R mRNA levels in hRPE cells. Expression levels of MC1R, MITF and Sp-1 mRNAs were studied after silencing of MITF-A and Sp-1 in hRPE cells. The data is presented as mean ± SD (n = 4) relative to cells transfected with non-specific control siRNA (=100 %). One-tailed *t*-test with Bonferroni correction; ∗*p* < 0.05, ∗∗*p* < 0.01 and ∗∗∗*p* < 0.001.

To verify the contribution of Sp1 and MITF transcription factors in regulation of *MC1R* expression, we silenced the expression of Sp1 and the main isoform of MITF in the RPE (MITF-A) [14,15] by RNA interference (Fig. 3) in primary hRPE cells that display many *in vivo*-like characteristics of RPE including melanin synthesis [34]. After exposure to MITF-A siRNA, transcript levels for *MC1R, MITF* and *Sp1* were decreased to 65 %, 11 % and 79 %, respectively of controls (*i.e*., cells transfected with the negative control siRNA). Exposure to Sp1-specific siRNA decreased *MC1R, MITF* and *Sp1* mRNAs to 52 %, 53 % and 27 %, respectively of control levels. Simultaneous silencing of both transcription factors decreased *MC1R, MITF* and *Sp1* mRNA levels even further to 27 %, 4 % and 14 %, respectively compared to controls. These data reveal that both MITF-A and Sp1 are required for *MC1R* expression.

## 4 Discussion

Although the defects in ocular pigmentation have been suggested as a risk factor for AMD, the genetic variations in pigmentation-related genes associated with developing wet AMD have remained unclear. This study was conducted in a retrospective manner by using variables and genomic DNA samples collected earlier. Unfortunately, no data of ocular melanin levels from the study participants was available. Furthermore, separation of melanin in the RPE and in the choroid is not straightforward [39]. Our results are in line with previous studies verifying the role of age and smoking as common risk factors for wet AMD [40]. Furthermore, associations between the complement factor H (*CFH*) and age-related maculopathy susceptibility 2 (*ARMS2*) gene variants and wet AMD have been consistently reported in European populations, including those analyzed in our study [[41], [42], [43], [44], [45], [46]]. Our association analysis yielded two significant variants with the risk of wet AMD in a Finnish case-control cohort. Despite the small sample numbers, the variant *rs*1407995 in the *DCT* gene intron significantly increased the risk of wet AMD, even when adjusted for age and sex. In addition, the variant *rs*3212351 in the *MC1R* gene was associated with wet AMD, although this did not survive similar age and gender adjustments. Interestingly, the SNP *rs*1407995 showed no association with wet AMD in other European replication cohorts. However, the rs3212351 variant data suggests a protective role in the Polish cohort while increasing the incidence of wet AMD in the Finnish population. It is known that the genetic heritage of Finns differs from other European populations [47]. Modern European populations represent a composite of ancestral European haplogroups shaped by migration, colonial expansion, and ethnic admixture. The impact of these multiple layers of genetic mixing is particularly evident in the UK and the analyzed Central European populations, whereas the Finnish population has remained comparatively isolated to the present day [48,49]. Exceptionally homogenous genetic background of Finns is due to combination of geographic isolation and cascade of historical genetic bottleneck events. This together with environmental selection of pigmentation genes and pigmentation traits could at least in part explain our population-specific findings [50].

Although we did not investigate functionality of the *rs*1407995 variant in the *DCT* intron, prior studies have shown this SNP to strongly affect human skin, hair and eye color [50,51]. Experimental models indicate that DCT mutations can affect pigment biosynthesis by increasing the pheomelanin and reducing eumelanin levels [52], as DCT catalyzes formation of the eumelanin intermediate 5,6-dihydroxyindole-2-carboxylic acid (DHICA).

Also, Frudakis et al. [53] and Valenzuela et al. [54] demonstrated association between several *DCT* and *MC1R* gene variants and different iris pigmentation phenotypes, including the DCT variant rs1407995. Interestingly, Valenzuela and colleagues also investigated the rs3212351 variant but could not show any association with iris pigmentation in their study population, which was recruited from students at the University of Arizona. The *MC1R* gene is highly polymorphic among Europeans. Its promoter variants associate with differences in pigmentation and pheomelanin phenotype with red hair and fair skin [18,55]. Transcriptional control of *MC1R* has mainly been studied in melanoma cells [13,38], but much less is known about pigmented cells of the eye. Moro and colleagues showed the functionality of proximal Sp1 (**S** at −109/-104 bp) and MITF (**M1** at −116/-111 bp) sites (shown in Fig. 2) for *MC1R* promoter activity in melanoma cells [37]. However, the deletion variant *rs*3212351 in distal MITF site was present in their promoter construct so that the effect of this **M2** site at −1236/-1231 bp for MC1R activity could not be ascertained. Because several *TYR* family genes are dissimilarly regulated between the RPE and melanocytes [15,56,57], it is likely that different *cis*-regulatory elements and variants therein also control *MC1R* transcription between these two developmentally distinct cell types.

We show here, for the first time, that the two *MC1R* promoter variants are functional and decrease *MC1R* activity in RPE cells. The SNP rs3212351 with a 2-bp deletion disrupts the MITF binding site and decreases the promoter activity slightly in RPE cells, whereas the proximal SNP rs3212360 at the Sp1 site attenuates promotor activity by half. In the genotyping assay validation, the SNP rs3212360 appeared monomorphic and was not therefore included in the association analysis. However, based on the high linkage disequilibrium and importance of Sp1 to MC1R expression in melanocytes [38], we chose to study its function in RPE cells beside the SNP rs3212351. Importantly, our RNA interference experiments in primary RPE cells showed that both transcription factors, MITF and Sp1 play a significant role in the regulation of endogenous MC1R, and that simultaneous knockdown of both factors resulted in a strong silencing of MC1R expression by 73 %.

**Fig. 4:**
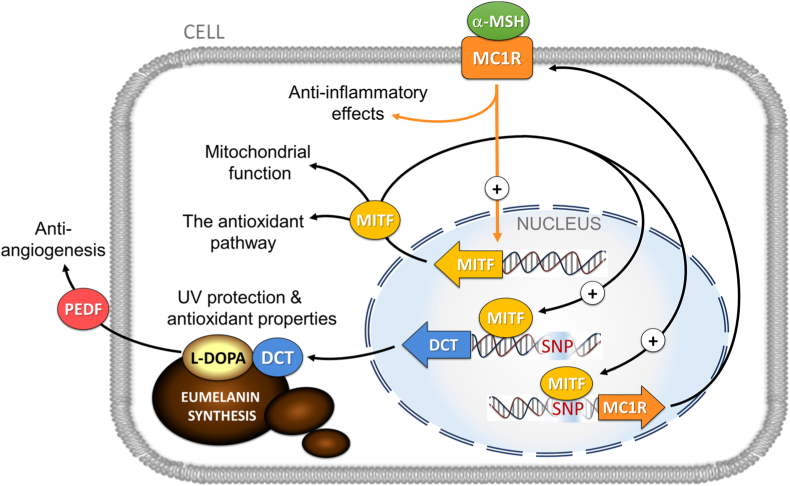
Known interactions of the MC1R-MITF pathway and DCT depicting critical regulatory loops involved in l-DOPA and melanin synthesis in RPE cells. The recently described associations with cellular antioxidant and immune defense mechanisms, mitochondrial function as well as PEDF-mediated anti-angiogenic actions are also shown. Associated risk variants rs1407995 and rs3212351 are indicated (SNP in red) in the *DCT* and *MC1R* genes, respectively.

Genetic traits with variations in *DCT* and *MC1R* genes can affect levels of cellular eumelanin, its precursor l-DOPA and finally the cellular eumelanin/pheomelanin ratio [18,52]. Disturbance in the melanin synthesis pathway with decreased eumelanin and elevated pheomelanin levels can disturb the cellular defense mechanisms (Fig. 4). Individuals with lighter iris, choroid and RPE pigmentation are thought to be less protected against solar radiation. Pheomelanin may have harmful pro-oxidant properties as shown by Mitra et al., [58] who reported increased oxidative damage in pheomelanin-abundant cells even in the absence of UV irradiation. Eumelanin functions as an endogenous antioxidant and a sink for metals and exogenous substances such as drugs [11,34]. The MC1R pathway also protects RPE cells from oxidative stress via the Akt-mTOR and nuclear factor erythroid 2-related factor 2 (NFE2L2) pathways [59,60]. Moreover, by interacting with the peroxisome proliferator-activated receptor-gamma coactivator-1α (PGC-1α), MITF plays a role in cellular pigment formation and mitochondrial biogenesis [61,62]. Loss of the PGC-1α coactivator contributed to RPE damage in a mouse model [63].

Interestingly, MC1R also has a role in the regulation of ocular inflammation [64]. *In vivo* retinal degeneration models also suggest a requirement for an intact αMSH signaling [65,66]. Acting via MC1R, αMSH contributes to ocular immunity as a strong suppressor of cytokine levels and inflammation [67].

While eumelanin has antioxidant properties, its precursor l-DOPA is known to have a significant role in retinal development as well as anti-angiogenic properties against pathogenic processes such as choroidal neovascularization. The RPE-derived l-DOPA activates secretion of pigment epithelium-derived factor (PEDF) [68] which is a potent endogenous anti-angiogenic factor in the eye [69]. Treatment with l-DOPA is correlated with some positive outcomes for wet AMD patients [70]. Collectively, these findings underscore the significance of pigmentation-related gene expression to support proper balance of RPE pigmentation.

## 5 Conclusions

In conclusion, our findings show that functional polymorphisms in human *DCT* and *MC1R* genes increase the risk of wet AMD in Finnish cohort. The *DCT* variant rs1407995 has been shown to decrease levels of ocular melanin pigment whereas the rs3212351 variant affect the expression of human *MC1R* gene in RPE cells, and thereby both variants can compromise homeostasis in the eye.

## Availability of data and materials

Datasets are available from the corresponding author upon reasonable request.

## Contributions

Conceptualization, Mika Reinisalo, Kai Kaarniranta and Paavo Honkakoski; Data curation, Seppo Helisalmi, Mikko Liukkonen and Helen Griffiths; Formal analysis, Seppo Helisalmi and Mikko Liukkonen; Funding acquisition, Arto Urtti, Kai Kaarniranta and Paavo Honkakoski; Investigation, Mika Reinisalo, Seppo Helisalmi, Ali Koskela, Jenni Küblbeck, Maija Mutikainen, Angela Cree, Helen Griffiths, Andras Papp, Sanna Seitsonen, Ilkka J.R. Immonen, Mateusz Winiarczyk, Miklos Resch and J. Arjuna Ratnayaka; Resources, Sanna Seitsonen, Ilkka J.R. Immonen, Hilkka Soininen, Arto Urtti, Mikko Hiltunen, Mateusz Winiarczyk, Miklos Resch, Andrew J Lotery, Kai Kaarniranta and Paavo Honkakoski; Supervision, Kai Kaarniranta and Paavo Honkakoski; Writing – original draft, Mika Reinisalo, Seppo Helisalmi, Ali Koskela, Jenni Küblbeck, Kai Kaarniranta and Paavo Honkakoski; Writing – review & editing, Mika Reinisalo, Seppo Helisalmi, Ali Koskela, Mikko Liukkonen, Mateusz Winiarczyk, Miklos Resch, J. Arjuna Ratnayaka, Andrew J Lotery, Kai Kaarniranta and Paavo Honkakoski.

## Ethics approval and consent to participate

The study was conducted in accordance with the Declaration of Helsinki. The Finnish study cohort was approved by the Ethics Committee of the Kuopio University Hospital (approval number 42/2014). The Polish cohort was approved by the Bioethical Committee of Medical University of Lublin approved the study (declaration number KE-0254/238/2015). In Hungary, the present study was approved by the Hungarian Medical Research Council Committee of Science and Research Ethics (Approval No. 15028-2/2017/EKU). In the UK, this study was approved by Southwest Hampshire Local Research Ethics Committee (374/02/t and 150/03/t).

## Consent for publication

Informed consent was obtained from all subjects involved in the study.

## Funding

This research was funded by the Finnish Funding Agency for Technology and Innovation. 10.13039/100005622Health Research Council of the 10.13039/501100002341Academy of Finland (grant numbers 296840. 333302, Gene Cell Nano Flagship). the 10.13039/501100013511Finnish Eye Foundation. the 10.13039/501100004092Kuopio University Hospital VTR grant (5503770). the Sigrid Juselius Foundation and the Päivikki and Sakari Sohlberg Foundation (all to Kai Kaarniranta) and by 10.13039/100005622Health Research Council of the 10.13039/501100002341Academy of Finland (Paavo Honkakoski. grant number 40440). 10.13039/501100002341Academy of Finland, PROFI5 (Seppo Helisalmi, grant number 325 022). 10.13039/100029627AJL was supported at the 10.13039/501100000739University of Southampton by funding from The 10.13039/100010269Wellcome Trust (076169/A). American Health Assistance Foundation (M2007110). Macula Vision Research Foundation. TFC Frost Charitable Trust. Brian Mercer Charitable Trust. Macular Society. Hobart Trust and the Gift of Sight appeal.

## Declaration of competing interest

The authors declare that they have no known competing financial interests or personal relationships that could have appeared to influence the work reported in this paper.

## Data Availability

Data will be made available on request.

## Abbreviations

AKT: Protein kinase B
AMD: Age-related Macular Degeneration
αMSH: α-melanocyte stimulating hormone
BMI: Body mass index
CI95: 95 % confidence interval
DCT: Dopachrome tautomerase
DHICA: 5,6-dihydroxyindole-2-carboxylic acid
eQTL: expression quantitative trait loci
GWAS: Genome-wide association studies
l-DOPA: L-3,4-dihydroxyphenylalanine
MC1R: Melanocortin-1 receptor
MITF: Microphthalmia-associated transcription factor
mTOR: Mammalian target of rapamycin
NFE2L2: Nuclear factor erythroid 2-related factor 2
OCT: Optical coherent tomography
OR: Odds ratio
OTX2: Orthodenticle homeobox-2
PEDF: Pigment epithelium-derived factor
PEI25: Polyethyleneimine 25
PGC-1α: Peroxisome proliferator-activated receptor-gamma coactivator-1α
RPE: Retinal pigment epithelium
SNP: Single Nucleotide Polymorphism
Sp1: Specificity protein 1
TYR: Tyrosinase
TYRP1: Tyrosinase-related protein 1
