# Supplementary material for: Functional polymorphisms in pigmentation-related genes MC1R and DCT display population-specific association with wet age-related macular degeneration
Source: Biochem Biophys Rep. 2026 Feb 6;45:102477. doi: 10.1016/j.bbrep.2026.102477 (PMC12905695; doi:10.1016/j.bbrep.2026.102477)
Supplement: Multimedia component 1 [file mmc1.pdf]

Supplemental material to:

Functional polymorphisms in pigmentation-related MC1R and DCT genes display population-specific association with wet age-related macular degeneration

Mika Reinisalo*, Seppo Helisalmi, Ali Koskela, Jenni Küblbeck, Mikko Liukkonen, Maija Mutikainen, Angela J Cree, Helen Griffiths, Andras Papp, Sanna Seitsonen, Ilkka Immonen, Hilkka Soininen, Arto Urtti, Mikko Hiltunen, Mateusz Winiarczyk, Miklos Resch, J. Arjuna Ratnayaka, Andrew J Lotery, Kai Kaarniranta, Paavo Honkakoski

*Corresponding author: Mika Reinisalo, School of Pharmacy, Faculty of Health Sciences, University of Eastern Finland, P.O.Box 1627, FI-70211 Kuopio, Finland.
E-mail: mika.reinisalo@uef.fi

Contents:

Supplemental Table S1. Main characteristics of the study populations.

Supplemental Table S2. Single nucleotide polymorphisms (SNPs) in genes associated with melanin synthesis studied in Finnish cohort.

Supplemental Table S3. Primers for the analysis of mRNA expression and site-directed mutagenesis.

Supplemental Table S4. Association of SNPs in pigmentation genes to wet AMD in the Finnish cohort using age- and sex -adjusted or crude recessive genetic models.

Supplemental Table S5. Association of SNPs in pigmentation genes to wet AMD in a Finnish cohort using crude or age and gender adjusted additive and dominant genetic models.

Supplemental Figure S1. Forest plots representing genotype frequencies and association of the MC1R variant rs3212351 to wet AMD in Polish, Hungarian, Finnish and UK cohorts.

Supplemental Figure S2. Forest plots representing genotype frequencies and association of the DCT variant rs1407995 to wet AMD in Polish, Hungarian, Finnish and UK cohorts.

Supplemental Table S6. Finnish cohort: Binary logistic regression analysis of the associations between SNP rs1407995, each variable and risk of wet AMD.

Supplemental Table S7. UK cohort: Binary logistic regression analysis of the associations between SNP rs1407995, each variable and risk of wet AMD.

Supplemental Table S8. Finnish cohort: Binary logistic regression analysis of the associations between SNP rs3212351, each variable and risk of wet AMD.

Supplemental Table S9. Finnish cohort: Binary logistic regression analysis of the associations between SNP rs3212351, each variable and risk of wet AMD.

Supplemental Table S1. Main characteristics of the study populations.

**Age**				
Cohort	(n)	Median	Range	*p*
Finland	Control (167)	75	56-94	0.000
	AMD (168)	80	50-100	
United Kingdom	Control (636)	68	49-91	0.000
	AMD (274)	80	33-94	
Poland	Control (113)	72	52-94	0.007
	AMD (124)	76	43-90	
Hungary	Control (43)	71	50-88	0.001
	AMD (209)	76	51-91	
**Sex**				
Cohort		Control (n)	AMD (n)	*p*
Finland	Male	55	60	0.592
	Female	112	108	
United Kingdom	Male	276	106	0.187
	Female	360	168	
Poland	Male	44	45	0.674
	Female	69	79	
Hungary	Male	15	85	0.480
	Female	28	124	
**Smoking habits**				
Cohort		Control (n)	AMD (n)	*p*
Finland	Non-Smoker	99	91	0.025
	Ex-Smoker	17	14	
	Regular Smoker	6	19	
United Kingdom	Non-Smoker	273	88	0.000
	Ex-Smoker	288	139	
	Regular Smoker	61	47	
**BMI**				
Cohort		Median	Range	*p*
Finland	Control	25.0	16-40	0.398
	AMD	25.0	19-58	
United Kingdom	Control	26.0	16-55	0.836
	AMD	25.5	14-43	
**Total**		Control (n)	AMD (n)	*p*
Total	n = 1734	959	775	
Age, Median [Range]		71,45 [48-94]	78, 67 [33-100]	0.000
Sex	Male	390	296	0.295
	Female	569	479	

n = number of subjects. Variables, age and body max index (BMI) were determined as continuous variables (range). Significance (*p*) in age and BMI between study groups were calculated using Mann-Whitney *U* test. Gender and smoking habits were analyzed with Chi-square test of independence. The level of statistical significance was set at $p < 0.05$. AMD = wet form of age-related macular degeneration.

Supplemental Table S2. Single nucleotide polymorphisms (SNPs) in genes associated with melanin synthesis studied in Finnish cohort.

SNP ID*	Allele Frequency Fin	Allele Frequency non-Fin Eur	Gene locus	Gene	Gene region	Polymorphism**
rs1427424	0.5373	0.4690	3p13	***MITF***	promoter	G > A
rs9869047	0.2355	0.2282				G > A
rs1036053	0.5917	0.6276			intron	T > C
rs7623486	0.1477	0.1851			intron	G > A
rs2762458	0.5820	0.5884	9p23	***TYRP1***	promoter	G > A
rs2762461	0.6265	0.6032			intron	G > T
rs10809828	0.2016	0.2262			intron	A > G
rs10830236	0.3238	0.3270	11q14.3	***TYR***	promoter	C > T
rs5021654	0.4312	0.3933			promoter	C > G
rs12791412	0.2700	0.2922			intron	A > G
rs2000553	0.4330	0.3943			intron	G > A
rs7924589	0.3946	0.3822			intron	G > A
rs10741305	0.5671	0.4845			intron	C > T
rs591260	0.5635	0.4818			intron	C > A
rs1126809	0.1587	0.2791			coding	CGA>CAA (R>Q)
rs10830253	0.3094	0.3116			intron	T > G
rs9524504	0.4196	0.4008	13q32.1	***DCT***	promoter	C > T
rs1407995	0.7825 (T > C)<br>0.0000 (T > A)	0.8151 (T > C)<br>0.00001 (T > A)			intron	T > C
rs928110	0.4475	0.4722	14q22.3	***OTX2***	promoter	G > T
rs1123285	0.3574	0.3363			intron	G > C
rs698015	0.6472	0.6405			intron	G > A
rs171978	0.0532	0.0966			3'-UTR	G > A
rs198241	0.8441	0.8476			3'-UTR	C > T
rs3212351	0.4348	0.3129	16q24.3	***MC1R***	promoter	AT > del
rs3212371	0.0807	0.1118			3'-UTR	G > A
rs7191944	0.0000 (C > A)<br>0.4344 (C > G)<br>0.0000 (C > T)	0.00001 (C > A)<br>0.2895 (C > G)<br>0.0000 (C > T)			3'-UTR	C > A/G

*ID = the single nucleotide polymorphism identifier in the NCBI SNP database (https://www.ncbi.nlm.nih.gov/snp/), 3′-UTR = the 3′ untranslated region. Frequencies taken from gnomAD, DATASET: gnomAD v3.1.2, GRCh38. **Polymorphism (wt>SNP)

Supplemental Table S3. Primers for the analysis of mRNA expression and site-directed mutagenesis.

MC1R mRNA (NM_002386, intronless gene)

forward 5´-GCAGCAGCTGGACAATGTC-3´

reverse 5´-GGGTCACGATGCTGTGGTAG-3

MITF mRNA (NM_198159, AB061771, NM_198177, NM_000248; exon 2/exon 3)

forward 5´- TGGCTATGCTTACGCTTAACTCC-3´

reverse 5´- ATACAGGACGCTCGTGAATGTG-3

Sp1 mRNA (NM_138473.2; exon 5/exon 6)

forward 5′-CACGTTCGGATGAGCTACAGAG-3′

reverse 5′-TCTTGATATGTTTTGACAGGTGGTC-3′

MITF rs3212351 -1233/-1232 deletion = CACATG → CAC--G

Sense primer: 5'-taatgtcctcacacac--ggaggtggcttgtgag-3'

Sp1 rs3212360 -105 C>T mutation = CCGCCC → CCGC**T**C

Sense primer: 5'-gcatgtggccgc**T**ctcagtgggagg-3'

Primers for Taqman analysis were designed by using OLIGO (Molecular Biology Insights, Inc., Cascade, CO) and BLAST (NCBI) programs to ensure their specificity. Complementary antisense primers for mutagenesis are not shown. Mutated nucleotides are shown with capital letters and MITF and Sp1 binding sites are doubly underlined. In the human *MC1R* sequence (NCBI accession number AF263461), the putative transcription start site is located at nucleotide 8698 (numbering here as +1).

Supplemental Table S4. Association of SNPs in pigmentation genes to wet AMD in the Finnish cohort using age- and sex -adjusted or crude recessive genetic models.

	Recessive Model					
	Age and sex adjusted			Crude		
**SNP**	**Sig.**	**OR**	**CI95**	**Sig.**	**OR**	**CI95**
rs1427424	0.607	0.867	(0.504-1.492)	0.540	0.850	(0.507-1.427)
rs9869047	0.691	0.841	(0.357-1.980)	0.421	0.713	(0.313-1.624)
rs1036053	0.846	1.068	(0.547-2.085)	0.623	1.174	(0.620-2.224)
rs7623486	0.135	0.353	(0.090-1.383)	0.084	0.310	(0.082-1.168)
rs2762458	0.319	0.726	(0.387-1.363)	0.404	0.774	(0.424-1.412)
rs2762461	0.316	0.676	(0.314-1.453)	0.562	0.805	(0.387-1.675)
rs10809828	0.202	2.489	(0.613-10.107)	0.162	2.614	(0.681-10.043)
rs10830236	0.767	0.898	(0.441-1.829)	0.393	0.743	(0.376-1.468)
rs5021654	0.155	0.661	(0.374-1.169)	0.081	0.615	(0.356-1.062)
rs12791412	0.788	1.110	(0.518-2.380)	0.833	0.924	(0.445-1.921)
rs2000553	0.564	0.838	(0.460-1.527)	0.411	0.787	(0.445-1.393)
rs7924589	0.568	0.834	(0.448-1.554)	0.377	0.764	(0.421-1.388)
rs10741305	0.273	0.732	(0.419-1.279)	0.453	0.816	(0.479-1.388)
rs591260	0.425	0.795	(0.453-1.396)	0.683	0.894	(0.524-1.527)
rs1126809	0.825	1.127	(0.391-3.250)	0.970	0.981	(0.359-2.680)
rs10830253	0.691	0.854	(0.391-1.861)	0.429	0.741	(0.352-1.560)
**rs1407995**	**0.018**	**4.514**	**(1.300-15.669)**	**0.043**	**3.250**	**(1.036-10.195)**
rs9524504	0.145	0.610	(0.313-1.187)	0.366	0.750	(0.403-1.398)
rs198241	0.862	0.896	(0.262-3.063)	0.965	0.975	(0.308-3.088)
rs171978	0.640	0.511	(0.031-8.528)	0.960	0.932	(0.058-15.034)
rs698015	0.665	0.870	(0.464-1.633)	0.707	0.891	(0.489-1.624)
rs1123285	0.741	1.106	(0.608-2.013)	0.673	1.131	(0.639-2.000)
rs928110	0.486	1.220	(0.697-2.138)	0.543	1.179	(0.693-2.005)
**rs3212351**	0.093	1.613	(0.923-2.819)	**0.048**	**1.714**	**(1.004-2.924)**
rs3212371	NA	NA	(NA-NA)	NA	NA	(NA-NA)
rs7191944	0.369	1.299	(0.734-2.298)	0.274	1.357	(0.785-2.345)

SNP = single nucleotide polymorphism, Sig. = significance (p<0.05 in bold), OR = odds ratio, CI95 = confidence interval at 95% confidence level. The *rs*1407995 and rs3212351 variants are highlighted in bold.

Supplemental Table S5. Association of SNPs in pigmentation genes to wet AMD in a Finnish cohort using crude or age and gender adjusted additive and dominant genetic models.

	Additive Model						Dominant Model					
	Age and sex adjusted			Crude			Age and sex adjusted			Crude		
**SNP**	**Sig.**	**Exp(B)**	**CI95**	**Sig.**	**Exp(B)**	**CI95**	**Sig.**	**Exp(B)**	**CI95**	**Sig.**	**Exp(B)**	**CI95**
rs1427424	0.312	0.848	(0.615-1.168)	0.328	0.859	(0.633-1.165)	0.259	0.744	(0.446-1.243)	0.324	0.783	(0.481-1.274)
rs9869047	0.499	0.882	(0.612-1.270)	0.219	0.804	(0.568-1.139)	0.516	0.857	(0.537-1.366)	0.255	0.773	(0.496-1.205)
rs1036053	0.357	1.179	(0.831-1.672)	0.334	1.179	(0.844-1.646)	0.251	1.331	(0.817-2.171)	0.321	1.266	(0.795-2.016)
rs7623486	0.620	0.899	(0.591-1.368)	0.374	0.833	(0.557-1.246)	0.958	1.014	(0.615-1.671)	0.759	0.928	(0.576-1.496)
rs2762458	0.384	0.865	(0.624-1.199)	0.413	0.878	(0.642-1.199)	0.613	0.886	(0.553-1.418)	0.579	0.880	(0.562-1.380)
rs2762461	0.382	0.855	(0.602-1.214)	0.492	0.889	(0.636-1.243)	0.584	0.879	(0.553-1.396)	0.578	0.882	(0.567-1.373)
rs10809828	0.802	0.946	(0.615-1.457)	0.973	1.007	(0.669-1.516)	0.416	0.810	(0.487-1.347)	0.588	0.875	(0.540-1.419)
rs10830236	0.891	0.976	(0.693-1.375)	0.545	0.905	(0.654-1.251)	0.993	1.002	(0.627-1.601)	0.789	0.941	(0.605-1.464)
rs5021654	0.471	0.891	(0.650-1.221)	0.282	0.848	(0.628-1.145)	0.906	1.029	(0.635-1.670)	0.875	0.964	(0.609-1.525)
rs12791412	0.843	1.036	(0.731-1.468)	0.841	0.967	(0.694-1.347)	0.920	1.024	(0.640-1.639)	0.888	0.969	(0.620-1.512)
rs2000553	0.776	1.048	(0.758-1.450)	0.975	0.995	(0.731-1.354)	0.374	1.246	(0.767-2.022)	0.538	1.156	(0.730-1.830)
rs7924589	0.991	1.002	(0.720-1.394)	0.649	0.930	(0.679-1.273)	0.645	1.120	(0.691-1.817)	0.986	1.004	(0.635-1.586)
rs10741305	0.850	0.969	(0.701-1.339)	0.842	1.032	(0.758-1.404)	0.483	1.199	(0.723-1.988)	0.321	1.277	(0.788-2.070)
rs591260	0.784	1.047	(0.753-1.455)	0.493	1.116	(0.815-1.527)	0.245	1.359	(0.810-2.278)	0.147	1.441	(0.879-2.362)
rs1126809	0.747	0.936	(0.625-1.401)	0.623	0.908	(0.619-1.333)	0.611	0.877	(0.529-1.453)	0.551	0.864	(0.534-1.397)
rs10830253	0.736	0.941	(0.661-1.340)	0.713	0.939	(0.671-1.313)	0.836	0.952	(0.597-1.518)	0.990	0.997	(0.640-1.553)
**rs1407995**	**0.038**	**1.537**	**(1.023-2.309)**	0.175	1.298	(0.890-1.893)	0.164	1.414	(0.868-2.304)	0.497	1.173	(0.740-1.859)
rs9524504	0.428	0.875	(0.628-1.218)	0.598	0.919	(0.673-1.257)	0.933	0.980	(0.615-1.563)	0.920	0.978	(0.627-1.524)
rs198241	0.331	1.240	(0.803-1.914)	0.226	1.289	(0.855-1.943)	0.216	1.388	(0.826-2.330)	0.139	1,450	(0.886-2.372)
rs171978	0.831	0.924	(0.446-1.914)	0.851	0.935	(0.463-1.887)	0.915	0.957	(0.427-2.144)	0.846	0.926	(0.426-2.013)
rs698015	0.852	0.970	(0.703-1.337)	0.961	0.992	(0.730-1.349)	0.960	1.012	(0.634-1.615)	0.835	1.049	(0.671-1.640)
rs1123285	0.942	1.011	(0.742-1.379)	0.678	1.065	(0.792-1.431)	0.882	0.965	(0.606-1.537)	0.768	1.069	(0.686-1.666)
rs928110	0.980	1.004	(0.729-1.383)	0.884	1.023	(0.754-1.388)	0.557	0.860	(0.520-1.423)	0.747	0.924	(0.571-1.495)
rs3212351	0.466	1.130	(0.814-1.567)	0.501	1.113	(0.815-1.519)	0.663	0.890	(0.527-1.504)	0.417	0.813	(0.493-1.340)
rs3212371	0.368	1.391	(0.678-2.853)	0.315	1.417	(0.718-2.796)	0.368	1.391	(0.678-2.853)	0.315	1.417	(0.718-2.796)
rs7191944	0.484	1.121	(0.814-1.545)	0.476	1.118	(0.823-1.520)	0.767	1.077	(0.659-1.760)	0.885	1.036	(0.646-1.659)

SNP = single nucleotide polymorphism, Sig. = significance ($p<0.05$ in bold), Exp(B) = exponentiation of the B coefficient (odds ratio), CI95 = confidence interval at 95% confidence level.

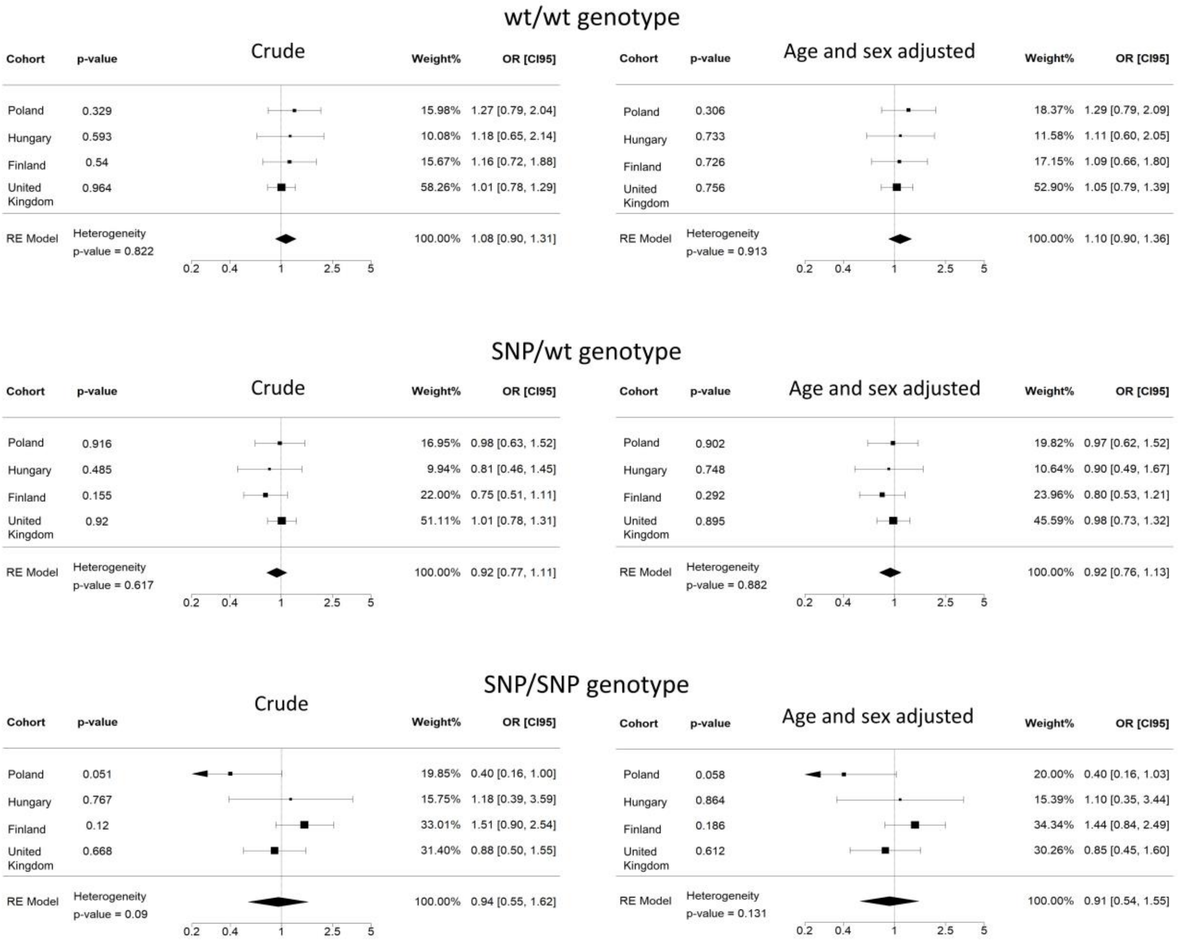

Supplemental Figure S1. Forest plots representing genotype frequencies and association of the MC1R variant rs3212351 to wet AMD in Polish, Hungarian, Finnish and UK cohorts. Crude and adjusted odds ratio (OR) with 95% confidence intervals (CI95) and p-values are shown.

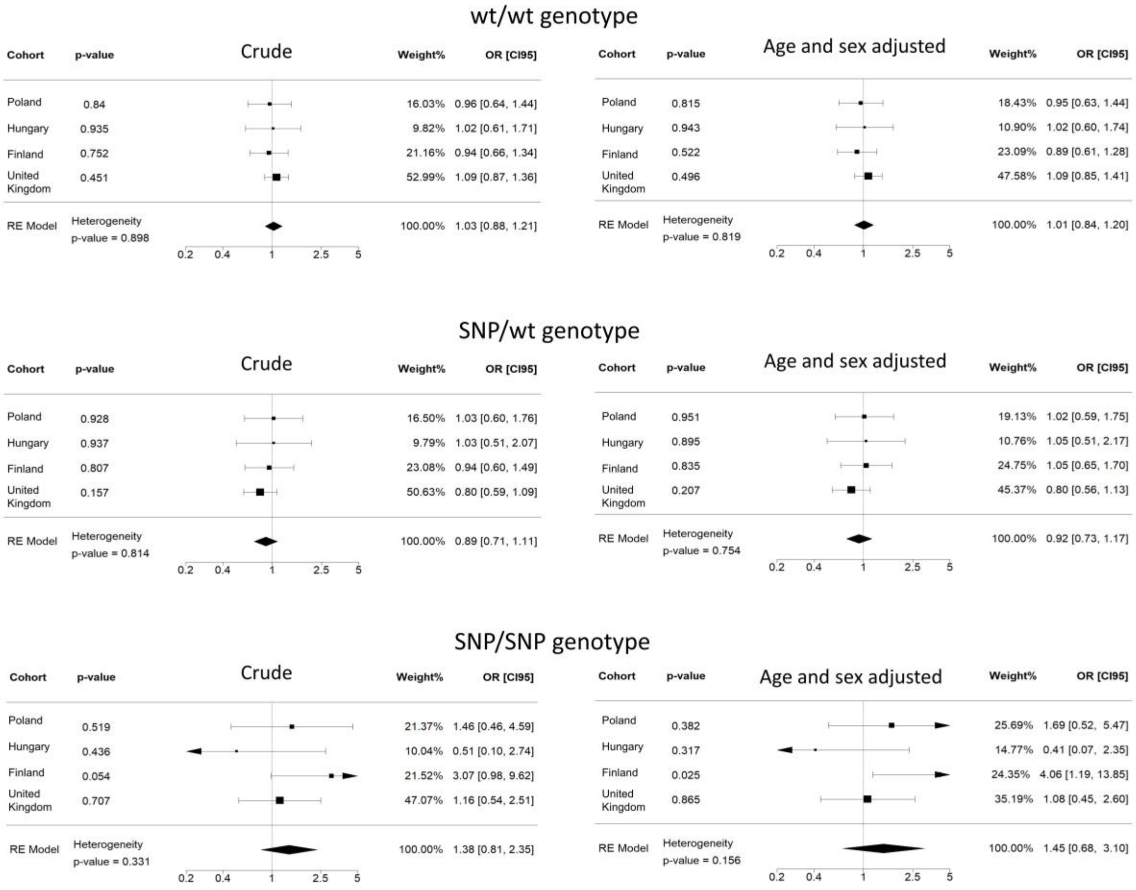

Supplemental Figure S2. Forest plots representing genotype frequencies and association of the DCT variant rs1407995 to wet AMD in Polish, Hungarian, Finnish and UK cohorts. Crude and adjusted odds ratio (OR) with 95% confidence intervals (CI95) and p-values are shown.

Supplemental Table S6. Finnish cohort: Binary logistic regression analysis of the associations between SNP rs1407995, each variable and risk of wet AMD.

	Variable	B	S.E.	Wald	df	Sig.	Exp(B)	95% C.I.for EXP(B) Lower	Upper
Step 1[a]	Sex(1)	-.092	.432	.046	1	.831	.912	.391	2.124
	Age	.118	.026	21.094	1	.000	1.125	1.070	1.183
	Smoking_status			10.488	2	.005			
	Smoking_status(1)	-.318	.529	.361	1	.548	.727	.258	2.053
	Smoking_status(2)	1.702	.613	7.712	1	.005	5.484	1.650	18.230
	BMI	.060	.032	3.446	1	.063	1.061	.997	1.130
	rs1407995	.522	.267	3.829	1	.050	1.685	.999	2.843
	Constant	-10.769	2.264	22.625	1	.000	.000		

	Variable	B	S.E.	Wald	df	Sig.	Exp(B)	95% C.I.for EXP(B) Lower	Upper
Step 1[a]	Age	.117	.025	21.922	1	.000	1.124	1.070	1.180
	Smoking_status			11.236	2	.004			
	Smoking_status(1)	-.249	.421	.351	1	.554	.779	.341	1.779
	Smoking_status(2)	1.762	.547	10.379	1	.001	5.823	1.994	17.006
	BMI	.059	.032	3.409	1	.065	1.061	.996	1.129
	rs1407995	.519	.267	3.796	1	.051	1.681	.997	2.834
	Constant	-10.736	2.256	22.646	1	.000	.000		

	Variable	B	S.E.	Wald	df	Sig.	Exp(B)	95% C.I.for EXP(B) Lower	Upper
Step 1[a]	Age	.116	.024	23.871	1	.000	1.123	1.072	1.176
	Smoking_status			10.646	2	.005			
	Smoking_status(1)	-.361	.419	.740	1	.390	.697	.307	1.585
	Smoking_status(2)	1.646	.540	9.284	1	.002	5.188	1.799	14.959
	rs1407995	.562	.259	4.686	1	.030	1.753	1.055	2.915
	Constant	-9.037	1.821	24.619	1	.000	.000		

Supplemental Table S7. UK cohort: Binary logistic regression analysis of the associations between SNP rs1407995, each variable and risk of wet AMD.

	Variable	B	S.E.	Wald	df	Sig.	Exp(B)	95% C.I.for EXP(B) Lower	Upper
Step 1[a]	Sex(1)	.452	.248	3.322	1	.068	1.572	.966	2.557
	Age	.126	.016	62.804	1	.000	1.134	1.100	1.170
	Smoking_status			11.144	2	.004			
	Smoking_status(1)	-.211	.257	.679	1	.410	.809	.489	1.339
	Smoking_status(2)	.978	.368	7.067	1	.008	2.660	1.293	5.470
	BMI	.019	.024	.636	1	.425	1.019	.973	1.067
	rs1407995	-.179	.208	.735	1	.391	.836	.556	1.258
	Constant	-10.724	1.482	52.358	1	.000	.000		

	Variable	B	S.E.	Wald	df	Sig.	Exp(B)	95% C.I.for EXP(B) Lower	Upper
Step 1[a]	Sex(1)	.261	.179	2.123	1	.145	1.299	.914	1.846
	Age	.135	.011	148.708	1	.000	1.144	1.120	1.169
	Smoking_status			16.147	2	.000			
	Smoking_status(1)	.260	.192	1.835	1	.176	1.297	.890	1.891
	Smoking_status(2)	1.093	.273	15.982	1	.000	2.982	1.745	5.095
	rs1407995	-.186	.156	1.430	1	.232	.830	.612	1.126
	Constant	-11.186	.862	168.376	1	.000	.000		

	Variable	B	S.E.	Wald	df	Sig.	Exp(B)	95% C.I.for EXP(B) Lower	Upper
Step 1[a]	Age	.137	.011	153.050	1	.000	1.146	1.122	1.171
	Smoking_status			15.017	2	.001			
	Smoking_status(1)	.182	.184	.982	1	.322	1.200	.837	1.720
	Smoking_status(2)	1.024	.267	14.669	1	.000	2.785	1.649	4.703
	Constant	-11.173	.853	171.656	1	.000	.000		

Supplemental Table S8. Finnish cohort: Binary logistic regression analysis of the associations between SNP rs3212351, each variable and risk of wet AMD.

	Variable	B	S.E.	Wald	df	Sig.	Exp(B)	95% C.I.for EXP(B) Lower	Upper
Step 1[a]	Sex(1)	-.068	.427	.025	1	.873	.934	.405	2.157
	Age	.119	.026	20.880	1	.000	1.126	1.070	1.185
	Smoking_status			11.636	2	.003			
	Smoking_status(1)	-.308	.523	.347	1	.556	.735	.263	2.049
	Smoking_status(2)	2.064	.680	9.217	1	.002	7.878	2.078	29.863
	BMI	.056	.032	3.074	1	.080	1.058	.993	1.126
	rs3212351	-.152	.211	.517	1	.472	.859	.568	1.299
	Constant	-10.379	2.277	20.768	1	.000	.000		

	Variable	B	S.E.	Wald	df	Sig.	Exp(B)	95% C.I.for EXP(B) Lower	Upper
Step 1[a]	Age	.118	.025	21.935	1	.000	1.125	1.071	1.182
	Smoking_status			12.163	2	.002			
	Smoking_status(1)	-.259	.421	.377	1	.539	.772	.338	1.762
	Smoking_status(2)	2.107	.625	11.345	1	.001	8.221	2.413	28.013
	BMI	.056	.032	3.052	1	.081	1.057	.993	1.126
	rs3212351	-.151	.211	.512	1	.474	.860	.569	1.300
	Constant	-10.349	2.267	20.836	1	.000	.000		

	Variable	B	S.E.	Wald	df	Sig.	Exp(B)	95% C.I.for EXP(B) Lower	Upper
Step 1[a]	Age	.120	.025	24.065	1	.000	1.128	1.075	1.184
	Smoking_status			11.717	2	.003			
	Smoking_status(1)	-.209	.418	.251	1	.617	.811	.357	1.841
	Smoking_status(2)	1.792	.539	11.055	1	.001	6.003	2.087	17.268
	BMI	.066	.032	4.348	1	.037	1.068	1.004	1.137
	Constant	-11.051	2.222	24.726	1	.000	.000		

Supplemental Table S9. Finnish cohort: Binary logistic regression analysis of the associations between SNP rs3212351, each variable and risk of wet AMD.

	Variable	B	S.E.	Wald	df	Sig.	Exp(B)	95% C.I.for EXP(B) Lower	Upper
Step 1[a]	Sex(1)	.474	.249	3.631	1	.057	1.606	.987	2.615
	Age	.126	.016	62.709	1	.000	1.134	1.099	1.170
	Smoking_status			11.697	2	.003			
	Smoking_status(1)	-.189	.257	.543	1	.461	.828	.501	1.369
	Smoking_status(2)	1.029	.369	7.774	1	.005	2.798	1.357	5.767
	BMI	.018	.024	.587	1	.443	1.018	.972	1.067
	rs3212351	.207	.181	1.316	1	.251	1.230	.863	1.753
	Constant	-10.897	1.481	54.151	1	.000	.000		

	Variable	B	S.E.	Wald	df	Sig.	Exp(B)	95% C.I.for EXP(B) Lower	Upper
Step 1[a]	Sex(1)	.275	.180	2.323	1	.127	1.316	.924	1.875
	Age	.136	.011	150.213	1	.000	1.146	1.121	1.171
	Smoking_status			17.535	2	.000			
	Smoking_status(1)	.275	.192	2.043	1	.153	1.317	.903	1.920
	Smoking_status(2)	1.147	.275	17.369	1	.000	3.148	1.836	5.398
	rs3212351	-.049	.136	.129	1	.720	.952	.730	1.243
	Constant	-11.323	.868	170.128	1	.000	.000		

	Variable	B	S.E.	Wald	df	Sig.	Exp(B)	95% C.I.for EXP(B) Lower	Upper
Step 1[a]	Age	.137	.011	153.050	1	.000	1.146	1.122	1.171
	Smoking_status			15.017	2	.001			
	Smoking_status(1)	.182	.184	.982	1	.322	1.200	.837	1.720
	Smoking_status(2)	1.024	.267	14.669	1	.000	2.785	1.649	4.703
	Constant	-11.173	.853	171.656	1	.000	.000		
